# Comparative Analysis of the Fatty Acid Profiles of Hypoallergenic Veterinary Diets, Commercial Dog Foods, and Oral Fatty Acid Supplements Available on the Hungarian Market

**DOI:** 10.3390/vetsci13101099

**Published:** 2026-10-16

**Authors:** Zsombor Tóth, Hedvig Fébel, László Wágner, Barbara Buránszki, Andrea Lajos, Kata Vida, Noémi Tarpataki

**Affiliations:** 1 Department of Internal Medicine, University of Veterinary Medicine, István u. 2, H-1078 Budapest, Hungary; toth.zsombor.tamas@univet.hu (Z.T.); tarpataki.noemi@univet.hu (N.T.); 2 Department of Obstetrics and Food Animal Medicine Clinic, University of Veterinary Medicine, István u. 2, H-1078 Budapest, Hungary; 3 Department of Animal Nutrition and Nutritional Physiology, Georgikon Campus, Hungarian University of Agriculture and Life Sciences (MATE), Deák Ferenc u. 16, H-8360 Keszthely, Hungary; wagner.laszlo@uni-mate.hu; 4 Pillevet Veterinary Practice, Dózsa György út 114, H-2134 Sződ, Hungary; drburanszki.barbara@gmail.com (B.B.); andi.lajos@gmail.com (A.L.); 5 Diósdvet Veterinary Practice, Sashegyi út 10, H-2049 Diósd, Hungary; vida.kata02@gmail.com

**Keywords:** omega-3 fatty acids, eicosapentaenoic acid, docosahexaenoic acid, canine atopic dermatitis, hypoallergenic diet, commercial dog food, gas chromatography, n-6/n-3 ratio, fatty acid supplement

## Abstract

Polyunsaturated fatty acids (PUFAs), particularly the long-chain n-3 PUFAs (eicosapentaenoic acid (EPA) and docosahexaenoic acid (DHA)), are integral to the dietary management of canine atopic dermatitis (CAD). However, the fatty acid profiles of commercially available canine products on Hungarian markets have not been systematically characterised, and the present study aimed to address this gap. A total of 35 products selected from those available on the Hungarian market at the time of sampling were analysed by gas chromatography with flame ionisation detection (GC-FID): 8 hypoallergenic veterinary diets (HVD), 22 commercial dog foods (CD), and 5 oral fatty acid supplements (FS). FS1–FS4 are complementary feeds under Regulation (EC) No 767/2009. FS5 is a food supplement under Directive 2002/46/EC. FS5 was included for comparative reference only. Fatty acid composition is expressed as % of total peak area. The n-6/n-3 ratio differed substantially across categories (HVD mean 6.16, range 2.65–10.71; CD mean 9.94, range 2.68–18.16; FS1–FS4 mean 18.08, range 0.29–63.49 (FS5 excluded as human product)). Combined % of EPA + DHA was lower in dietary products (HVD mean 0.94%, range 0.16–2.10%; CD mean 0.19%, range 0.09–0.34%) than in marine-based supplements (FS3 7.52%, FS4 24.19%). Alpha-linolenic acid (ALA) was the predominant n-3 fatty acid in 29 of 30 dietary products. Pronounced inter-product heterogeneity was observed among supplements, including one plant-based product with an n-6/n-3 ratio of 63.49. These findings indicate that commercial and hypoallergenic diets contain low proportion of EPA, and that marine-based oral supplementation provided substantially higher proportions of EPA and DHA than any dietary product evaluated.

## Simple Summary

Dogs with atopic dermatitis—a common, chronic inflammatory skin disease—frequently receive dietary supplementation with omega-3 polyunsaturated fatty acids, as part of their management. These beneficial fatty acids, especially eicosapentaenoic acid and docosahexaenoic acid, help reduce inflammation and improve quality of life. However, the actual fatty acid composition of commercial dog foods and oral supplements sold in Hungary has not been systematically characterised. We analysed 35 products—8 hypoallergenic veterinary diets, 22 standard commercial dog foods, and 5 oral fatty acid supplements (four veterinary and one human, the latter included for comparative reference)—available on the Hungarian market using gas chromatography with flame ionisation detection, a standard analytical method. Our results show that commercial dog foods contain very low proportions of eicosapentaenoic acid, and that the ratio of omega-6 to omega-3 fatty acids is unfavourable in the majority of standard products. Hypoallergenic diets show a better average omega-6 to omega-3 balance, but considerable product-level variability exists. Marine-based veterinary oral supplements provided the highest proportions of clinically relevant eicosapentaenoic acid and docosahexaenoic acid. This study frames the utility of the findings in terms of category-level guidance.

## 1. Introduction

Canine atopic dermatitis (CAD) is a chronic inflammatory skin disorder in dogs, associated with IgE-mediated hypersensitivity. CAD is characterised by pruritus, erythema, and recurrent secondary cutaneous infections in genetically predisposed individuals [1,2,3]. While the exact prevalence in the general canine population is unknown, atopic skin disease is estimated to affect 10–15% of the dog population in industrialised countries, with an increasing incidence reported in recent decades [1,4]. The pathogenesis of CAD is multifactorial, involving genetic predisposition, immunological dysregulation, skin barrier dysfunction, microbiome changes and environmental triggers [5,6].

Disruption of epidermal barrier integrity is a defining feature of CAD pathogenesis. Histological and ultrastructural studies have demonstrated that the stratum corneum of atopic dogs exhibits abnormal intercellular lipid lamellae, reduced ceramide content, and increased transepidermal water loss compared with healthy skin [7,8,9,10,11,12]. These changes are thought to promote allergen penetration and sustain cutaneous inflammation. Dietary lipids, and in particular polyunsaturated fatty acids (PUFAs), are integral structural components of the epidermal lipid matrix. Linoleic acid (LA, 18:2n-6) is esterified into omega-hydroxy ceramides—key structural lipids of the stratum corneum—and its adequate dietary supply is essential for maintaining a functional epidermal barrier [13,14,15,16,17].

Beyond their structural role in the skin, long-chain n-3 PUFAs—primarily eicosapentaenoic acid (EPA, 20:5n-3) and docosahexaenoic acid (DHA, 22:6n-3)—exert systemic anti-inflammatory effects by competitively inhibiting arachidonic acid (AA, 20:4n-6) as a substrate for cyclooxygenase and lipoxygenase pathways, thereby shifting eicosanoid production towards less potent pro-inflammatory mediators [18,19,20,21,22]. In dogs, n-3 PUFA supplementation reduces the synthesis of leukotriene B4 (LTB4) in skin and neutrophils [23,24,25,26,27], and clinical trials have shown that dietary n-3 enrichment reduces pruritus, decreases reliance on glucocorticoids, and attenuates clinical signs of CAD [28,29,30,31]. The beneficial effect appears to be dependent on both the absolute n-3 supply and the n-6/n-3 ratio of the diet, with a ratio between 5:1 and 10:1 recommended for modulating cutaneous inflammation [23,28,32,33].

The n-3 PUFA alpha-linolenic acid (ALA, 18:3n-3) is the most abundant n-3 fatty acid in many plant-derived oils commonly used in pet food formulation. However, dogs have a limited capacity to elongate and desaturate ALA to EPA and DHA via the hepatic delta-6-desaturase pathway, and dietary ALA supplementation does not reliably increase circulating EPA or DHA to levels comparable to direct marine supplementation [33,34,35,36,37,38]. It has therefore been argued that preformed long-chain n-3 PUFAs from marine sources are the effective dietary form for dogs, and that ALA, although not without value, should not be regarded as an adequate surrogate for EPA and DHA in the clinical management of CAD [24,32,34,35,39,40,41].

Evidence from clinical trials and expert recommendations consistently supports increasing the dietary intake of essential fatty acids—in particular LA, ALA, EPA, and DHA—as part of the chronic management of CAD [28,31,33,38,39]. Supplementation may be achieved via topical application [42,43,44,45,46], oral fatty acid supplements [28,29,40,42,47,48,49,50], or through complete commercial diets [30,51,52], including hypoallergenic formulations prescribed as part of dietary elimination trials for suspected adverse food reactions [53,54,55,56]. Hypoallergenic diets are especially relevant in this context: their protein source is restricted to novel proteins (e.g., insect meal [57,58,59,60], or venison [61,62]) or hydrolysed proteins (e.g., hydrolysed poultry or salmon [63,64,65,66]) to minimise antigenic stimulation [54,67], but this formulation constraint necessarily limits available fat sources and may substantially influence the final lipid composition of the product. Despite their widespread clinical use, the actual fatty acid profiles of hypoallergenic diets have not been systematically characterised or compared with those of standard commercial dog foods.

Beyond dermatology, n-3 PUFAs have shown clinical benefit in canine osteoarthritis [68,69], cardiac arrhythmia and heart failure [70,71], chronic kidney disease [72], and neurodevelopment [36,73]. The nutritional importance of EPA and DHA across multiple organ systems reinforces the need for accurate characterisation of the fatty acid supply potential of commercially available products. Product label information on fatty acid content is not uniformly provided or standardised, and discrepancies between declared and analytically determined values have been previously documented [74,75,76]. The Omega-3 Index—the EPA + DHA content of erythrocyte membranes expressed as a percentage of total fatty acids—has been proposed as a biomarker of n-3 status in dogs, and its application may help bridge the gap between dietary product composition and actual tissue n-3 incorporation [77,78,79].

To the authors’ knowledge, only two previous studies have systematically characterised the fatty acid profiles of commercial canine dietary products by gas chromatography: Ahlstrøm et al. [75] examined fatty acid composition of Norwegian commercial dog foods in 2004, and Martinez and McDonald [76] analysed Australian veterinary diets, supplements and fish oil capsules in 2021. Both reported negligible EPA and DHA concentrations in dietary products, with ALA as the predominant n-3 source. Neither study included Central or Eastern European market products, and neither directly compared hypoallergenic and standard commercial diets within the same analytical framework. The present study therefore aimed to determine and compare the fatty acid profiles of hypoallergenic veterinary diets, standard commercial dog foods, and oral fatty acid supplements available on the Hungarian market, using GC-FID as the reference analytical method, with specific attention to parameters directly relevant to the clinical management of CAD: the n-6/n-3 ratio and the relative proportion of preformed long-chain n-3 PUFAs (EPA and DHA). The GC-FID analysis was performed to characterise the fatty acid profile of the dog foods and to determine the relative proportion of the individual fatty acids. Accordingly, the results are presented as relative area percentage data.

## 2. Materials and Methods

### 2.1. Product Selection

A total of 35 commercially available canine products were selected for analysis, comprising three categories: (i) hypoallergenic veterinary diets (HVD, *n* = 8), defined as products marketed and dispensed for dietary elimination trials or dermatological indications, containing novel or hydrolysed protein sources; (ii) standard commercial dog foods (CD, *n* = 22), representing brands widely available in Hungarian retail and veterinary distribution channels; and (iii) oral fatty acid supplements (FS, *n* = 5), including products marketed for canine oral administration. One human product (FS5) was included for comparative reference only. Hypoallergenic veterinary diets were selected to represent the range of prescription formulations available through veterinary distribution channels in Hungary at the time of sampling. Commercial dog foods were selected from products widely available in Hungarian retail and veterinary distribution channels, with the aim of representing a range of commonly marketed brands and product types; no formal sampling frame based on sales volume or market share was applied. Oral fatty acid supplements (FS1–FS4) represent the complementary veterinary feed products and commercially available in Hungary at the time of sampling. All products were purchased between 10 and 12 February 2025 in intact, sealed packaging and stored in a dark, cool environment until analysis. Sample preparation and GC-FID analysis commenced on 13 February 2025 and were completed over approximately one month. The interval between sample purchase and completion of all analytical procedures was approximately 30 days, a relatively short period that helped minimise the risk of sample deterioration and lipid oxidation. Label-declared crude fat content (as-fed basis) was extracted from each product’s packaging at the time of purchase and are reported in Supplementary Table S1. For pet foods, crude fat declaration is mandatory under Regulation (EC) No 767/2009 [80]; for the human oral fatty acid supplement (FS5), the corresponding nutritional declaration is governed by Regulation (EU) No 1169/2011 [81]. The conclusions of this study should be interpreted in the context of the Hungarian market and the specific products sampled at that time. A single sample per product was analysed; batch-to-batch variability was not assessed. All products are identified by anonymous codes (HVD1–HVD8, CD1–CD22, FS1–FS5) throughout this paper; product identities are withheld to preclude commercially biased interpretation of the findings.

### 2.2. Sample Preparation and FAME Synthesis

For dry dog food (HVD1–HVD6; CD11, CD12), a representative portion of approximately 200 g per package was finely ground using a laboratory mill to achieve a uniform powder. For wet canned food (HVD7, HVD8; CD1–CD10 and CD13–CD22), the entire contents of the cans (approx. 400 g) were thoroughly blended and homogenised into a uniform paste using a laboratory homogenizer. Liquid oil supplements (FS1–FS5) were sampled following ISO 5555 [82] and prepared according to the ISO 661 [83] protocol using chemically inert, stainless steel equipment. Sampling and preparation of the oil supplements used for the treatment of canine dermatitis were performed in strict accordance with international standards. Representative laboratory samples were collected following the guidelines of ISO 5555, using chemically inert, stainless steel equipment to prevent contamination or degradation. Prior to analytical testing and administration, the samples were prepared and homogenised according to the ISO 661 protocol. Solid or cloudy oil matrices were gently liquefied in a water bath at 50 °C (or 10 °C above their melting point) and thoroughly blended to ensure uniform distribution of the active components. For capsule supplements, the liquid contents of the capsules were pooled and mixed extensively to ensure uniformity before sampling.

Total lipids were extracted from homogenised samples (5 g aliquot of dry or wet diets or 1 g of oil supplement) using the classical Folch method (chloroform:methanol, 2:1, *v*/*v*). To prevent lipid oxidation, the organic solvent was evaporated to dryness under a gentle stream of nitrogen gas. From the resulting pure, isolated lipid fraction, an analytical aliquot of approximately 25 mg was weighed into a 1.8 mL reaction vial. Saponification was performed by adding 400 µL of 1 N NaOH in methanol; vials were sealed and incubated at 100 °C for 15 min, then cooled to room temperature. Subsequently, 600 µL of boron trifluoride–methanol (BF_3_–MeOH) was added, vials were resealed and incubated at 60 °C for 10 min. After cooling, 400 µL of n-hexane and 200 µL of saturated NaCl solution were added; samples were vortex-mixed and centrifuged 3000× *g* for 15 min. An aliquot of the upper organic phase was transferred to a GC vial containing anhydrous Na_2_SO_4_ and stored at −20 °C until analysis. The preparation of FAMEs was conducted according to the guidelines of ISO 12966-2:2017 [84].

### 2.3. Gas Chromatographic Analysis

Separation was carried out via capillary gas chromatography (GLC) in compliance with the ISO 12966-4:2015 standard [85]. Analyses were performed on a TRACE 2000 gas chromatograph (Thermo Finnigan, Waltham, MA, USA) equipped with an Omegawax 320 fused silica capillary column (30 m × 0.32 mm ID × 0.25 μm film thickness; Supelco, Bellefonte, PA, USA). The analysis was performed using a 55 min temperature-programmed oven profile. The oven temperature was initially set at 90 °C and held for 2 min, followed by an increase to 120 °C at a rate of 5 °C/min and held for 4 min followed by an increase to 200 °C at a rate of 5 °C/min. The temperature was then maintained at 200 °C for 12 min, increased to 260 °C at 6 °C/min, and finally held at 260 °C for 5 min. The split/splitless inlet was operated in split mode (split ratio 1:100) at 250 °C; injection volume was 1 µL. Helium carrier gas was maintained at a constant inlet pressure of 65 kPa. The FID detector was operated at 260 °C with air (350 mL/min), hydrogen (35 mL/min), and make-up gas (30 mL/min) flows. Peak integration parameters: peak width 1 s, threshold 1, minimum area 50,000. Individual fatty acids were identified by retention time comparison with an analytical standard used for relative determination of fatty acids (Supelco Polyunsaturated Fatty Acid (PUFA) No. 2, Animal Source, Methyl Esters; Cat. No. 47015-U; Sigma-Aldrich/Supelco, Bellefonte, PA, USA). Standard mixture contains the following FAMEs: C14:0, C16:0, C16:1n7, C18:0, C18:1n9, C18:1n7, C18:2n6, C18:3n6, C18:3n3, C20:0, C20:1n9, C20:3n6, C20:4n6, C20:5n3, C22:4n6, C22:5n3, C22:6n3. Lauric acid (C12:0) is not included among the components listed for the PUFA No. 2 standard. The laboratory utilised pure, individual FAME reference standard for this specific fatty acid and mixed with standard mixture. Relative fatty acid composition was calculated from chromatographic peak areas and data are expressed as percentage of total peak area. Analyses were performed at the analytical laboratory of Hungarian University of Agriculture and Life Sciences (Keszthely, Hungary). The sensitivity of the gas chromatographic method was characterised using the institutional LOD and LOQ values routinely applied to the GC-FID system. The LOD and LOQ for individual fatty acid methyl esters were set at 0.005% and 0.015% of total fatty acids, respectively.

### 2.4. Data Analysis

The relative proportions of individual fatty acids were summed within each fatty acid class: saturated fatty acids (SFA: C12:0, C14:0, C16:0, C18:0), monounsaturated fatty acids (MUFA: C16:1n-7, C18:1n-9, C18:1n7, C20:1n-9), n-6 PUFAs (LA, GLA, C20:4n6, C22:4n-6), and n-3 PUFAs (ALA, EPA, DPA (docosapentaenoic acid), DHA). The n-6/n-3 ratio was calculated individually for each product. As each product was measured as a single sample without analytical or biological replication, descriptive statistics (mean, standard deviation and range) are reported throughout and no inferential statistical testing was applied. Standard deviations reflect inter-product variability within each category and do not represent analytical measurement uncertainty. All calculations were performed in Microsoft Excel (version 2019, Microsoft Corp., Redmond, WA, USA). All cross-category comparisons in the Results and Discussion are descriptive in nature; they reflect the specific products sampled and should not be interpreted as statistically significant differences between product categories.

## 3. Results

### 3.1. Fatty Acid Profiles of Hypoallergenic Veterinary Diets (HVD1–HVD8)

The fatty acid composition of the eight hypoallergenic veterinary diets is presented in Table 1. Detailed results for individual fatty acids are presented in Supplementary Table S2. SFA was the dominant fraction in three of eight products (HVD2, HVD3 and HVD6), with a category mean of 39.09 ± 8.19% (range: 32.55–54.00%); MUFA predominated in the remaining five products (HVD1, HVD4, HVD5, HVD7 and HVD8). Palmitic acid (C16:0) was the predominant SFA across most products; the exception was HVD3, in which lauric acid (C12:0) predominated (25.21%) and the high C12:0 proportion is consistent with copra oil (coconut oil) as the declared lipid source. HVD3 exhibited an unusually elevated proportion of GLA (3.94%) and C22:4n-6 (3.32%). Ingredient label review identified copra oil, soya oil, fish oil and animal fat as the declared fat sources of HVD3. The peaks assigned to GLA and C22:4n-6 could not be accounted for by any of the declared ingredients and are therefore considered unverified assignments.

MUFA constituted the second most abundant fraction (33.67 ± 9.52%, range: 16.46–44.98%), with oleic acid (C18:1n-9) dominant in all products. Total proportion of n-6 PUFA ranged from 9.85 to 24.17% (mean: 18.90%), with LA as the predominant n-6 component. Total n-3 PUFA ranged from 0.92 to 9.13% (mean: 3.99%), with ALA as the principal n-3 fatty acid in the products. Combined EPA + DHA ranged from 0.16 to 2.10%; the highest values were recorded in HVD6 (2.10%) and HVD4 (2.06%). The n-6/n-3 ratio ranged from 2.65 to 10.71 (6.16 ± 2.99). Three products (HVD5, HVD6, HVD8) had a ratio below 5:1; four (HVD1–HVD4) fell within 5–10:1; and one (HVD7) exceeded 10:1.

### 3.2. Fatty Acid Profiles of Commercial Dog Foods (CD1–CD22)

The fatty acid profiles of the 22 commercial diets are shown in Table 2. Detailed results for individual fatty acids are presented in Supplementary Table S2. SFA ranged from 24.19 to 59.05% (39.03 ± 8.58%) and MUFA from 25.34 to 52.69% (40.21 ± 6.27%), with oleic acid predominant in all products. Total n-6 PUFA ranged from 6.72 to 26.60% (14.46 ± 5.25%), with LA as the major component. Total proportion of n-3 PUFA was lower than in the hypoallergenic veterinary diet category (mean: 1.90%, range: 0.37–5.46%). ALA was the principal n-3 fatty acid, while EPA and DHA were present at trace proportions: mean EPA 0.10% (range: 0.01–0.30%) and mean DHA 0.09% (range: 0.03–0.16%). Combined EPA + DHA ranged from 0.10 to 0.34%, representing the lowest values across all three categories.

The n-6/n-3 ratio ranged from 2.68 to 18.16 (9.94 ± 4.59). Twelve of 22 products (55%) exceeded 10:1; five (CD9, CD11, CD12, CD13, CD15) showed ratios below 5:1; and five (CD3, CD10, CD14, CD18 and CD19) fell within 5–10:1. Products that included salmon or other marine species in the product name did not systematically differ from other commercial diets in EPA or DHA content.

### 3.3. Fatty Acid Profiles of Oral Fatty Acid Supplements (FS1–FS5)

The fatty acid composition of the five oral supplements is shown in Table 3. Detailed results for individual fatty acids are presented in Supplementary Table S2. FS5 is for human use and is included for comparative reference. Marked inter-product heterogeneity was observed. FS2 was characterised by a high proportion of LA (45.30%), reflecting its plant oil composition. FS1 also exhibited a predominance of LA (40.71%), but concurrently contained 1.01% EPA and 3.65% DHA (EPA + DHA: 4.66%), consistent with its declared formula which includes EPA and DHA alongside plant-derived fatty acids, indicating a mixed plant/marine oil composition. FS2 exhibited the highest n-6/n-3 ratio of all 35 products analysed (63.49) and low proportion of EPA + DHA (0.34%). In contrast, the marine-based veterinary supplements (FS3 and FS4) and the human supplement FS5 contained substantial proportions of EPA and DHA. FS4 showed the highest EPA (10.67%) and DHA (13.52%) of all products, with an n-6/n-3 ratio of 0.29. FS5 provided similarly elevated proportions of EPA + DHA (18.39%; n-6/n-3 ratio: 0.26). FS3 showed a mixed n-3 profile with both ALA (5.85%) and EPA + DHA (7.52%), and an n-6/n-3 ratio of 0.84. Mean EPA + DHA across veterinary supplements (FS1–FS4) was 9.18% (range: 0.34–24.19%). The highest percentage of unidentified fatty acid fraction of all 35 products was found in FS1 and FS2, 8.93% and 9.68%, respectively.

## 4. Discussion

### 4.1. n-6/n-3 Ratios and Their Clinical Relevance to CAD

The n-6/n-3 ratio is a widely used parameter in the dietary management of CAD, with a range of 5:1 to 10:1 considered to favour anti-inflammatory eicosanoid production in dogs [23,28,32,33]. Our findings reveal descriptive differences across product categories; no statistical comparisons were performed. Hypoallergenic veterinary diets (HVD) showed an average n-6/n-3 ratio of 6.16 (range: 2.65–10.71), placing four of eight products within the clinically recommended range, while commercial diets exhibited a mean of 9.94 (range: 2.68–18.16), with 55% exceeding 10:1. These results are consistent with the characterisations provided by Ahlstrøm et al. [75] and Martinez and McDonald [76], both of whom reported that standard commercial dog foods tend to have unfavourable n-6/n-3 ratios relative to veterinary formulations, and with broader evidence that Western-type pet food formulations are typically enriched in n-6 fatty acids from plant-derived fat sources.

However, the n-6/n-3 ratio alone is an insufficient descriptor of a diet’s anti-inflammatory potential. The biological activity of n-3 fatty acids in modulating inflammatory pathways depends on the molecular form in which they are supplied. Moreover, dogs have limited capacity to elongate and desaturate ALA to EPA and DHA, primarily due to low hepatic delta-6-desaturase activity, and this conversion is insufficient to raise tissue EPA and DHA concentrations to levels achievable through direct marine supplementation [30,34]. Consistent with this, Dominguez et al. [34] demonstrated that flaxseed oil supplementation, despite providing equivalent amounts of total n-3 PUFAs, failed to increase the omega-3 index in dogs, whereas krill oil (providing preformed EPA and DHA) produced a significant increase. Similarly, Stoeckel et al. [79] showed that incorporation of n-3 PUFAs into canine erythrocyte membranes—a recognised surrogate marker of tissue n-3 status—occurs within one week of dietary supplementation with marine oil-based diets, whereas the effect is minimal with ALA-based sources. These findings indicate that the source composition of n-3 fatty acids, not just the total n-3 content or n-6/n-3 ratio, determines the anti-inflammatory efficacy of a dietary intervention.

### 4.2. EPA and DHA Supply from Dietary Products: A Clinical Gap

In this study, ALA was the predominant n-3 fatty acid in 29 of 30 dietary products analysed; the single exception was CD20, in which the combined long-chain n-3 PUFA content (EPA + DPA + DHA: 0.28%) marginally exceeded ALA (0.27%), while EPA and DHA were present at trace proportions in both commercial diets (range 0.09–0.34%) and HVDs (range 0.16–2.10%). Earlier clinical trials demonstrating efficacy in CAD have typically employed supplementation doses providing 50–85 mg EPA + DHA per kg body weight per day [6,29,30]. Since the present study characterises fatty acid as a relative proportion rather than as absolute gravimetric quantities, direct dose calculations are not possible without independent knowledge of the total fat content and energy density of each product. The extent to which the observed EPA + DHA proportions could translate into clinically relevant intakes under real-world feeding conditions therefore cannot be determined from the present data alone [32].

The finding that products bearing salmon or marine ingredient names in the commercial diet category did not systematically provide higher proportion of EPA or DHA than other commercial foods is of direct clinical significance. Product naming and marketing claims may not reliably reflect the actual quantity of marine ingredients incorporated, and the contribution of fish meal—as opposed to fish oil—to the long-chain n-3 PUFA content of a kibble may be limited. Bauer [35] noted that individual long-chain n-3 fatty acid concentrations are not mandatory label declarations in pet food regulation, and that ingredient lists are often insufficient to estimate the EPA and DHA content of a diet without analytical verification. Our data support this concern and are consistent with previous observations [75,76].

### 4.3. Characteristics of Hypoallergenic Veterinary Diets

The eight hypoallergenic veterinary diets showed considerable intra-category heterogeneity in fatty acid composition. HVD3 was noteworthy for its exceptionally high proportion of lauric acid (C12:0) (25.21%), consistent with the inclusion of copra oil (coconut oil) in the formulation—a fat source characterised by high lauric acid (C12:0) content. Although the term ‘MCT’ is sometimes used loosely in veterinary nutrition, commercial MCT oils consist predominantly of caprylic (C8:0) and capric acid (C10:0); these short-chain fatty acids were not analysed and are therefore absent from the reported profile. The high C12:0 proportion in HVD3 is consistent with copra oil as the declared lipid source, rather than reflecting a methodological omission. This atypical SFA profile may have implications for skin lipid composition, given that lauric acid is not a physiological constituent of canine epidermal ceramides and is unlikely to contribute to restoration of the stratum corneum lipid lamellae in the same manner as LA-derived ceramides [12,14,16,17]. The products with the highest EPA + DHA proportion combined with the lowest n-6/n-3 ratio—compositional characteristics consistent with deliberate formulation with marine-sourced lipids—were HVD6 (EPA + DHA 2.10%; n-6/n-3 ratio 2.65) and HVD4 (EPA + DHA 2.06%; n-6/n-3 ratio 5.04). At the other extreme, HVD7—marketed as a veterinary dermatological diet—exhibited the lowest EPA + DHA proportion (0.16%) and the highest n-6/n-3 ratio (10.71) within the HVD category. This finding highlights that therapeutic product designation does not guarantee an analytically favourable fatty acid composition, and reinforces the need for independent analytical verification when selecting dietary products for the management of CAD.

### 4.4. Heterogeneity Among Supplements and Its Clinical Implications

The supplement category showed the greatest inter-product variability of any category examined, spanning more than 70-fold range in EPA + DHA content (0.34–24.19%) and a more than 200-fold range in n-6/n-3 ratio (0.29–63.49). FS2, a plant oil-based supplement dominated by LA (45.30%), exhibited an n-6/n-3 ratio of 63.49—the highest of all 35 products. Administration of FS2 to a CAD patient already receiving an n-6-rich commercial diet would further widen an already elevated n-6/n-3 ratio. While LA itself is an essential structural component of the stratum corneum and adequate dietary supply is important for epidermal barrier function [13,14,15,16,17], an n-6/n-3 ratio of 63.49 represents a marked imbalance between n-6 and n-3 substrates. On compositional grounds, this profile is inconsistent with the dietary targets recommended for the management of CAD [28,30]. Whether this substrate disproportion translates to adverse clinical outcomes cannot be determined from fatty acid profile data alone and remains to be evaluated in controlled intervention studies. It should be noted, however, that GLA (C18:3n-6) was detected in both FS2 (0.75%) and FS1 (1.85%). GLA may exert anti-inflammatory effects via its elongation to DGLA—a conversion demonstrated in vivo in dogs with atopic dermatitis [86] —and subsequent production of 15-HETrE, which inhibits 5-lipoxygenase-mediated leukotriene synthesis [87]. This pathway may partially offset the compositional n-6/n-3 imbalance, particularly for FS1, which additionally provides substantial EPA + DHA (4.66%). Nevertheless, this finding is relevant from a prescribing perspective, given that such products are available without veterinary prescription and may be self-selected by owners without professional guidance. Veterinarians should therefore specify not only whether to supplement, but which product type to recommend, on the basis of the individual patient’s dietary background.

In contrast, the two marine-based supplements (FS3, FS4) provided EPA + DHA concentrations substantially higher than any dietary product examined (7.52–24.19%), demonstrating that, among the products evaluated, concentrated marine oil preparations provided substantially higher proportions of preformed EPA + DHA than any dietary product examined. Whether this compositional advantage translates to superior clinical outcomes depends on absolute intake, bioavailability, and individual patient factors beyond the scope of the present analysis [34]. These data also show that DHA and EPA concentrations vary considerably even among marine supplements, likely reflecting differences in the source species, processing method, and manufacturing practices. In particular, the molecular form in which n-3 PUFAs are delivered may influence their bioavailability: Dyerberg et al. [88] demonstrated in a randomised human study that re-esterified triglycerides provided superior EPA + DHA bioavailability (124% relative to natural fish oil), whereas ethyl esters showed inferior absorption (73%), and free fatty acids did not differ significantly from natural triglycerides (91%). Whether these formulation-dependent differences in bioavailability translate to clinically meaningful differences in tissue n-3 incorporation in dogs remains to be established. FS5 (a human food supplement under Directive 2002/46/EC [89] ) included for comparative reference only, contained 18.39% EPA + DHA. This value falls between those of FS3 (7.52%) and FS4 (24.19%), but direct comparison with veterinary products is not appropriate: species-specific dosing, excipient safety in dogs, and regulatory status differ fundamentally. FS5 data are therefore reported descriptively and excluded from all category statistics.

### 4.5. Limitations

Several limitations of this study must be acknowledged. First, each product was analysed as a single sample without analytical replication or assessment of batch-to-batch variability. The results therefore represent the specific samples purchased between 10 and 12 February 2025, and may not fully characterise the fatty acid profile of a given product across production batches or over time. This is a meaningful limitation in the context of commercial pet food manufacturing, where batch-to-batch variability in lipid composition can be substantial. The original samples are no longer available, precluding retrospective analytical replication. Second, the product selection reflects market availability on the Hungarian market at the time of sampling and is not necessarily representative of the wider Central European, European, or global market. Proportional statements derived from this dataset (e.g., ‘55% of CD products exceeded an n-6/n-3 ratio of 10:1’) describe the composition of the specific products sampled and should not be extrapolated to the broader Hungarian or European market. Third, fatty acid stability following product opening was not assessed; however, Martinez and McDonald [76] found no significant change in FA content over 56 days under controlled conditions. Fourth, the study characterises the relative proportions of fatty acids of products but cannot predict achieved plasma or tissue EPA/DHA levels in individual patients, which depend on feeding amounts, background diet, body weight, and individual metabolic variation [41,88,90]. Fifth, the sum of reported fatty acid fractions (SFA + MUFA + Σn-6 + Σn-3) does not reach 100% in any product analysed. The relative proportions of the identified fatty acids were calculated from their chromatographic peak areas and expressed as percentages of the total chromatographic peak area. Consequently, the reported percentages may be influenced by the contribution of unidentified components. Sixth, the EPA values reported for HVD1, HVD4 and HVD6 should be interpreted with caution. The measured EPA:DHA ratios in these products (5.6:1, 16.2:1 and 13.0:1, respectively) are substantially higher than values typical of fish oil (approximately 1–1.5:1) and deviate markedly from manufacturer-declared fatty acid values. The reported EPA values for these three products therefore represent upper bounds rather than confirmed measurements, and inter-product EPA comparisons within the hydrolysed veterinary diet category should be made with this caveat in mind. Seventh, the unexpectedly low DHA values may be related to a matrix-, extraction- or integration-related loss in the diets. The low DHA values are presented as an unverified analytical observation.

## 5. Conclusions

This study provides the first systematic GC-FID characterisation of fatty acid profiles across hypoallergenic veterinary diets, commercial dog foods, and oral fatty acid supplements from the Hungarian market. The principal findings are as follows. Commercial dog foods showed uniformly low proportions of preformed EPA and DHA (mean 0.19%; range 0.09–0.34%), with 55% of products exceeding the upper bound of the recommended n-6/n-3 ratio range. Hypoallergenic veterinary diets show more favourable n-6/n-3 ratios as a category, but substantial intra-category variability exists, including at least one product with the lowest EPA + DHA proportion and highest n-6/n-3 ratio within the category despite its dermatological designation. Marine-based veterinary oral fatty acid supplements provided substantially higher proportions of preformed EPA + DHA than any dietary product evaluated; the human food supplement (FS5), included for comparative reference only, provided 18.39% EPA + DHA—a value between those of FS3 (7.52%) and FS4 (24.19%); direct comparison with veterinary products is not appropriate given differences in regulatory status, excipient safety, and species-specific dosing. The extreme heterogeneity observed among supplements—one product had an n-6/n-3 ratio of 63.49—highlights the importance of product-specific fatty acid information when making supplement recommendations, given that compositional characteristics vary widely and cannot be inferred from product category alone. These findings support routine analytical verification of fatty acid content in canine dietary products and emphasise the importance of product-specific supplement selection in the nutritional management of CAD. Future studies should incorporate analytical replication, temporal stability assessment, systematic label-accuracy evaluation, and investigation of the relationship between dietary fatty acid supply and achieved tissue EPA + DHA status in dogs with atopic dermatitis.

## Figures and Tables

**Table 1:** Fatty acid composition of hypoallergenic diets (HVD1–HVD8). Values are expressed as percentages of total peak area (%).

Product	SFA	MUFA	Σn-6	LA	GLA	AA	DTA	Σn-3	ALA	DPA	EPA	DHA	n-6/n-3
HVD1	34.11	36.87	21.02	20.27	0.17	0.49	0.09	3.01	2.01	0.07	0.79	0.14	6.98
HVD2	49.49	26.20	17.69	17.08	0.23	0.25	0.13	2.14	1.30	0.09	0.62	0.13	8.27
HVD3	54.00	16.46	23.15	14.09	3.94 *	1.80	3.32 *	2.61	1.70	0.11	0.66	0.14	8.87
HVD4	32.55	34.98	24.14	21.78	0.14	1.99	0.23	4.79	2.59	0.14	1.94	0.12	5.04
HVD5	32.96	40.19	17.45	17.19	0.03	0.07	0.16	4.49	4.01	0.12	0.28	0.08	3.89
HVD6	35.03	27.87	24.17	23.11	0.08	0.84	0.14	9.13	6.85	0.18	1.95	0.15 ^§^	2.65
HVD7	39.60	44.98	9.85	9.10	0.08	0.60	0.07	0.92	0.66	0.10	0.10	0.06 ^§^	10.71
HVD8	34.99	41.82	13.73	12.79	0.35	0.46	0.13	4.80	4.23	0.21	0.13	0.23	2.86
Mean	39.09	33.67	18.90	16.93	0.63	0.81	0.53	3.99	2.92	0.13	0.81	0.13	6.16
SD	8.19	9.52	5.21	4.77	1.34	0.71	1.13	2.50	2.02	0.05	0.74	0.05	2.99
Range	32.55–54.00	16.46–44.98	9.85–24.17	9.10–23.11	0.03–3.94	0.07–1.99	0.07–3.32	0.92–9.13	0.66–6.85	0.07–0.21	0.10–1.95	0.06–0.23	2.65–10.71

SD: Standard Deviation; SFA: saturated fatty acids; MUFA: monounsaturated fatty acids; Σn-6: total n-6 polyunsaturated fatty acids; LA: C18:2n-6 (linoleic acid); GLA: C18:3n-6 (γ-linolenic acid); AA: C20:4n-6 (arachidonic acid); DTA: C22:4n-6 docosatetraenoic acid; Σn-3: total n-3 polyunsaturated fatty acids; ALA: alpha-linolenic acid (C18:3n-3); DPA: C22:5n-3 (docosapentaenoic acid); EPA: eicosapentaenoic acid (C20:5n-3); DHA: docosahexaenoic acid (C22:6n-3). * Unverified peak assignment; no declared feed ingredient provides a clear source that would explain the presence of this fatty acid at the reported proportion. ^§^ Unverified analytical observations.

**Table 2:** Fatty acid composition of commercial dog foods (CD1–CD22). Values are expressed as percentages of total peak area (%).

Product	SFA	MUFA	Σn-6	LA	GLA	AA	DTA	Σn-3	ALA	DPA	EPA	DHA	n-6/n-3
CD1	31.85	44.62	18.08	17.55	0.07	0.42	0.04	1.67	1.34	0.08	0.20	0.05	10.83
CD2	45.82	38.90	9.81	8.82	0.11	0.84	0.04	0.77	0.62	0.06	0.06	0.03	12.74
CD3	59.05	25.34	9.83	8.87	0.26	0.58	0.12	1.09	0.82	0.11	0.08	0.08	9.02
CD4	36.69	44.66	13.38	12.49	0.11	0.72	0.06	1.18	0.96	0.07	0.10	0.05	11.34
CD5	46.85	38.01	9.67	8.97	0.09	0.49	0.12	0.54	0.32	0.06	0.09	0.07	17.91
CD6	50.66	32.02	11.94	8.14	0.31	3.47	0.02	1.05	0.87	0.08	0.01 †	0.09	11.37
CD7	35.01	39.09	19.54	18.27	0.15	1.07	0.05	1.42	1.17	0.11	0.04	0.10	13.76
CD8	46.38	29.85	18.64	17.72	0.14	0.63	0.15	1.40	1.20	0.07	0.05	0.08	13.31
CD9	41.88	41.84	9.70	8.37	0.08	1.19	0.06	2.56	2.13	0.09	0.30	0.04	3.79
CD10	24.19	52.69	15.78	14.66	0.11	0.80	0.21	3.03	2.58	0.11	0.21	0.13	5.21
CD11	39.98	35.92	16.60	15.87	0.10	0.49	0.14	3.38	2.96	0.13	0.18	0.11	4.91
CD12	38.82	40.40	12.84	12.52	0.07	0.16	0.09	3.17	2.89	0.13	0.08	0.07	4.05
CD13	28.12	39.63	22.67	21.88	0.18	0.48	0.13	5.46	4.99	0.14	0.21	0.12 ^§^	4.15
CD14	29.31	47.77	16.20	15.40	0.15	0.56	0.09	2.40	2.15	0.06	0.11	0.08	6.75
CD15	50.29	34.59	7.48	6.62	0.13	0.64	0.09	2.79	2.48	0.15	0.07	0.09	2.68
CD16	36.85	39.48	17.83	15.45	0.54	1.26	0.58	1.38	1.09	0.12	0.07	0.10	12.92
CD17	29.24	37.03	26.60	25.46	0.15	0.86	0.13	2.58	2.37	0.08	0.04	0.09	10.31
CD18	30.87	41.63	20.28	19.35	0.18	0.66	0.09	2.68	2.38	0.12	0.04	0.14	7.57
CD19	34.75	44.32	14.84	13.75	0.11	0.95	0.03	1.51	1.31	0.03	0.12	0.05	9.83
CD20	40.52	45.82	8.72	5.84	0.07	2.55	0.26	0.55	0.27	0.08	0.11	0.09	15.85
CD21	38.26	46.00	10.88	10.38	0.14	0.29	0.07	0.89	0.51	0.13	0.09	0.16	12.22
CD22	43.31	44.96	6.72	6.09	0.07	0.45	0.11	0.37	0.21	0.04	0.04	0.08	18.16
Mean	39.03	40.21	14.46	13.29	0.15	0.89	0.12	1.90	1.62	0.09	0.10	0.09	9.94
SD	8.58	6.27	5.25	5.39	0.11	0.75	0.12	1.22	1.16	0.03	0.07	0.03	4.59
Range	24.19–59.05	25.34–52.69	6.72– 26.60	5.84–25.46	0.07–0.54	0.16–3.47	0.02–0.58	0.37–5.46	0.21–4.99	0.03–0.15	0.01–0.30	0.03–0.16	2.68– 18.16

SD: Standard Deviation; SFA: saturated fatty acids; MUFA: monounsaturated fatty acids; Σn-6: total n-6 polyunsaturated fatty acids; LA: C18:2n-6 (linoleic acid); GLA: C18:3n-6 (γ-linolenic acid); AA: C20:4n-6 (arachidonic acid); DTA: C22:4n-6 docosatetraenoic acid; Σn-3: total n-3 polyunsaturated fatty acids; ALA: alpha-linolenic acid (C18:3n-3); DPA: C22:5n-3 (docosapentaenoic acid); EPA: eicosapentaenoic acid (C20:5n-3); DHA: docosahexaenoic acid (C22:6n-3); The category SD reflects the disproportionate influence of CD16 (0.58%); the remaining 21 products ranged from 0.02 to 0.26% (mean 0.09 ± 0.05). † Fatty acid value falling between the LOD (0.005%) and LOQ (0.015%) thresholds was fully detectable but should be interpreted as trace proportion due to the inherently higher measurement uncertainty below the quantification limit. ^§^ Unverified analytical observations.

**Table 3:** Fatty acid composition of oral fatty acid supplements (FS1–FS5). Values are expressed as percentages of total peak area (%).

Product	SFA	MUFA	n-6	LA	GLA	AA	DTA	n-3	ALA	DPA	EPA	DHA	n-6/n-3
FS1	15.58	26.96	42.95	40.71	1.85	0.30	0.09	5.58	0.77	0.15	1.01	3.65	7.70
FS2	13.99	29.25	46.35	45.30	0.75	0.21	0.09	0.73	0.25	0.14	0.13	0.21	63.49
FS3	19.78	49.12	12.44	10.45	0.49	0.96	0.54	14.88	5.85	1.51	3.24	4.28	0.84
FS4	25.53	34.26	8.11	2.43	2.16	2.57	0.95	28.31	1.89	2.23	10.67	13.52	0.29
FS5 *	22.57	47.69	5.22	4.52	0.16	0.28	0.26	20.47	0.58	1.50	12.00	6.39	0.26
Mean (FS1–FS4)	18.72	34.90	27.46	24.72	1.31	1.01	0.46	12.38	2.19	1.01	3.76	5.42	18.08
SD	5.16	9.96	19.97	21.45	0.82	1.09	0.38	12.14	2.53	0.90	4.79	5.69	30.46
Range (FS1–FS4)	13.99–25.53	26.96–49.12	8.11–46.35	2.43–45.30	0.49–2.16	0.21–2.57	0.09–0.95	0.73–28.31	0.25–5.85	0.14–2.23	0.13–10.67	0.21–13.52	0.29–63.49

SD: Standard Deviation; SFA: saturated fatty acids; MUFA: monounsaturated fatty acids; Σn-6: total n-6 polyunsaturated fatty acids; LA: C18:2n-6 (linoleic acid); GLA: C18:3n-6 (γ-linolenic acid); AA: C20:4n-6 (arachidonic acid); DTA: C22:4n-6 docosatetraenoic acid;; Σn-3: total n-3 polyunsaturated fatty acids; ALA: alpha-linolenic acid (C18:3n-3); DPA: C22:5n-3 (docosapentaenoic acid); EPA: eicosapentaenoic acid (C20:5n-3); DHA: docosahexaenoic acid (C22:6n-3). The high category mean and SD for DTA reflect the disproportionate influence of FS4 (0.95%); the remaining three veterinary products (FS1–FS3) ranged from 0.09 to 0.54% (mean 0.24 ± 0.24). * FS5 is a human food supplement under Directive 2002/46/EC; it is included for comparative reference only and excluded from all category statistics.

## Data Availability

The original contributions presented in this study are included in the article/Supplementary Material. Further inquiries can be directed to the corresponding author.

## Supplementary Materials

The following supporting information can be downloaded at https://www.mdpi.com/article/10.3390/vetsci13101099/s1.

## Abbreviations

The following abbreviations are used in this manuscript:

AA	Arachidonic acid (C20:4n-6)
ALA	Alpha-linolenic acid (C18:3n-3)
BF_3_–MeOH	Boron trifluoride–methanol
BW	Body weight
CAD	Canine atopic dermatitis
CD	Commercial dog food
DGLA	Dihomo-γ-linolenic acid (C20:3n-6)
DHA	Docosahexaenoic acid (C22:6n-3)
DPA	Docosapentaenoic acid (C22:5n-3)
EPA	Eicosapentaenoic acid (C20:5n-3)
FA	Fatty acid
FAME	Fatty acid methyl ester
FS	Oral fatty acid supplement
GC-FID	Gas chromatography with flame ionisation detection
GLA	Gamma-linolenic acid (C18:3n-6)
HVD	Hypoallergenic veterinary diet
IgE	Immunoglobulin E
LA	Linoleic acid (C18:2n-6)
LOD	Limit of detection
LOQ	Limit of quantification
LTB4	Leukotriene B4
MCT	Medium-chain triglyceride
MUFA	Monounsaturated fatty acid
PUFA	Polyunsaturated fatty acid
SFA	Saturated fatty acid
SD	Standard deviation
TEWL	Transepidermal water loss
15-HETrE	15-Hydroxyeicosatrienoic acid
