# Supplementary material for: Comparative Analysis of the Fatty Acid Profiles of Hypoallergenic Veterinary Diets, Commercial Dog Foods, and Oral Fatty Acid Supplements Available on the Hungarian Market
Source: Vet Sci. 2026 Oct 16;13(10):1099. doi: 10.3390/vetsci13101099 (PMC13709901; doi:10.3390/vetsci13101099)
Supplement: Supplementary file 1 [file vetsci-13-01099-s001.zip › Supplementary Table S1.pdf]

Supplementary Table S1. Product information for all 35 tested products

Code	Format	Protein source(s)	Protein source type	Declared fat source(s)	Fish oil present	Crude fat % (as-fed, declared)
**Hypoallergenic/Veterinary Diets (HVD, n = 8)**						
HVD1	dry	Hydrolysed fish protein + hydrolysed pork/poultry	Hydrolysed, multiple	Poultry fat	No	18.0
HVD2	dry	Hydrolysed soy + hydrolysed poultry liver	Hydrolysed, multiple	Fish oil	Yes	19.0
HVD3	dry	Feather hydrolysate (L-amino acids)	Hydrolysed, single	Fish oil, copra oil, soya oil, animal fat	Yes	16.5
HVD4	dry	Duck + hydrolysed poultry	Intact + hydrolysed, multiple	Fish oil	Yes	9.0
HVD5	dry	Hydrolysed soy protein	Hydrolysed, single	Coconut oil, rapeseed oil, soya oil, fish oil	Yes	11.0
HVD6	dry	Egg + rice protein	Intact, multiple	Fish oil (1.8%)	Yes	15.2
HVD7	wet	Pork liver, pork meat	Intact, multiple	Salmon oil	Yes	6.5
HVD8	wet	Pork (65%)	Intact, single	—	No	8.5
**Commercial Diets (CD, n = 22)**						
CD1	wet	Ostrich	Intact, single	Animal fat	No	5.2
CD2	wet	Goat	Intact, single	Animal fat	No	6.3
CD3	wet	Lamb	Intact, single	Animal fat	No	8.0
CD4	wet	Duck	Intact, single	Animal fat	No	7.5
CD5	wet	Buffalo	Intact, single	Animal fat	No	7.0
CD6	wet	Venison	Intact, single	Animal fat	No	7.0
CD7	wet	Chicken, liver	Intact, multiple	Animal fat	No	5.0
CD8	wet	Beef, chicken	Intact, multiple	Animal fat	No	5.0
CD9	wet	Heart, poultry liver	Intact, multiple	Animal fat	No	7.0
CD10	wet	Chicken	Intact, single	Animal fat	No	5.5
CD11	dry	Poultry	Intact, single	—	No	1.0
CD12	dry	Poultry	Intact, single	Fish oil	Yes	36.0
CD13	wet	Salmon, herring	Intact, multiple	Fish (salmon, herring)	Yes	4.0
CD14	wet	Rabbit, salmon	Intact, multiple	Fish (salmon)	Yes	4.0
CD15	wet	Beef (68%)	Intact, single	Sunflower oil	No	8.0
CD16	wet	Chicken (68%)	Intact, single	Sunflower oil	No	5.2
CD17	wet	Turkey (68%)	Intact, single	Sunflower oil	No	5.5
CD18	wet	Salmon (14%)	Intact, single	Fish (salmon)	Yes	6.0
CD19	wet	Salmon (10%) + meat/animal derivatives	Intact, multiple	Fish + animal derivatives	Yes	7.0
CD20	wet	Beef + meat/animal derivatives	Intact, multiple	Animal derivatives	No	7.0
CD21	wet	Meat/animal derivatives (40%) + fish/fish derivatives (20%, tuna 7%)	Intact, multiple	Animal + fish derivatives	Yes	8.0
CD22	wet	Duck (8%) + meat/animal derivatives	Intact, multiple	Animal derivatives	No	6.5
**Food Supplements (FS, n = 5)**						**Calculated fat content %†**
FS2	liquid supplement	—	—	Fish oil + plant oils	Yes	~75†
FS3	liquid supplement	—	—	Salmon oil (100%)	Yes	~99†
FS4	liquid supplement	—	—	Wild salmon oil	Yes	~99†
FS5	liquid supplement	—	—	Fish oil (anchovy, mackerel, sardine) + olive oil	Yes	~99†

† Calculated from declared fatty acid composition; crude fat not explicitly stated on label (supplement category).

Abbreviations: HVD, hypoallergenic/veterinary diet; CD, commercial diet; FS, food supplement.
