# Supplementary material for: Comparative Analysis of the Fatty Acid Profiles of Hypoallergenic Veterinary Diets, Commercial Dog Foods, and Oral Fatty Acid Supplements Available on the Hungarian Market
Source: Vet Sci. 2026 Oct 16;13(10):1099. doi: 10.3390/vetsci13101099 (PMC13709901; doi:10.3390/vetsci13101099)
Supplement: Supplementary file 1 [file vetsci-13-01099-s001.zip › Supplementary Table S2.pdf]

Supplementary Table S2. Individual fatty acid composition (% of total peak area) of hypoallergenic veterinary diets (HVD1–8), commercial dog foods (CD1–22), and oral fatty acid supplements (FS1–5).

	Hypoallergenic Veterinary Diets (HVD)								Commercial Dog Foods (CD)																						Fatty Acid Supplements (FS)				
**Fatty Acid**	**HVD1**	**HVD2**	**HVD3**	**HVD4**	**HVD5**	**HVD6**	**HVD7**	**HVD8**	**CD1**	**CD2**	**CD3**	**CD4**	**CD5**	**CD6**	**CD7**	**CD8**	**CD9**	**CD10**	**CD11**	**CD12**	**CD13**	**CD14**	**CD15**	**CD16**	**CD17**	**CD18**	**CD19**	**CD20**	**CD21**	**CD22**	**FS1**	**FS2**	**FS3**	**FS4**	**FS5***
**Saturated fatty acids (SFA)**																																			
C12:0 (lauric acid)	0.82	0.91	25.21	3.71	13.10	8.41	nd	nd	nd	nd	nd	nd	nd	nd	nd	nd	nd	nd	nd	nd	nd	nd	nd	nd	nd	nd	nd	nd	nd	nd	nd	nd	nd	nd	nd
C14:0 (myristic acid)	8.33	8.44	11.21	3.47	6.59	4.64	1.46	2.23	0.73	2.83	2.96	1.05	2.95	2.24	1.60	1.16	1.69	1.60	5.83	7.77	1.08	0.83	6.24	1.36	0.69	0.92	1.36	1.32	1.59	1.50	2.59	1.83	4.84	5.07	4.50
C16:0 (palmitic acid)	19.25	32.55	12.79	19.58	10.09	16.22	24.59	22.20	22.53	26.60	37.80	24.65	27.38	24.75	22.63	34.97	24.18	16.22	24.86	23.47	20.97	21.83	23.12	27.18	21.50	22.23	24.95	29.66	26.81	31.22	9.82	8.58	11.15	15.79	14.73
C18:0 (stearic acid)	5.71	7.59	4.79	5.79	3.18	5.76	13.55	10.56	8.59	16.39	18.29	10.99	16.52	23.67	10.78	10.25	16.01	6.37	9.29	7.58	6.07	6.65	20.93	8.31	7.05	7.72	8.44	9.54	9.86	10.59	3.17	3.58	3.79	4.67	3.34
**Monounsaturated fatty acids (MUFA)**																																			
C16:1n-7 (palmitoleic acid)	4.70	2.76	1.08	3.11	0.71	1.71	1.87	3.11	2.31	2.03	2.18	2.41	2.49	2.26	2.06	2.55	1.60	2.82	1.91	3.53	3.03	3.52	3.44	3.87	3.23	2.75	3.25	3.24	3.03	3.59	3.15	2.32	4.79	6.24	4.57
C18:1n-9 (oleic acid)	31.26	22.36	14.73	30.72	38.46	25.10	40.94	34.54	41.30	35.45	22.01	41.19	34.61	28.09	35.96	26.25	38.91	48.26	32.72	35.67	35.42	43.31	30.15	34.07	33.02	36.80	38.25	39.05	39.43	38.82	22.82	25.97	38.18	17.61	40.06
C18:1c11 (vaccenic acid)	0.51	0.68	0.43	0.62	0.52	0.70	1.26	2.02	0.48	0.77	0.72	0.43	0.49	1.13	0.56	0.58	0.81	1.06	0.89	0.76	0.58	0.51	0.81	1.19	0.53	1.30	1.38	1.88	1.92	1.61	0.50	0.51	0.91	1.12	1.67
C20:1n-9 (eicosenoic acid)	0.40	0.40	0.22	0.53	0.50	0.36	0.91	2.15	0.53	0.65	0.43	0.63	0.42	0.54	0.51	0.47	0.52	0.55	0.40	0.44	0.60	0.43	0.19	0.35	0.25	0.78	1.44	1.65	1.62	0.94	0.49	0.45	5.24	9.29	1.39
**n-6 Polyunsaturated fatty acids**																																			
C18:2n-6 (linoleic acid)	20.27	17.08	14.09	21.78	17.19	23.11	9.10	12.79	17.55	8.82	8.87	12.49	8.97	8.14	18.27	17.72	8.37	14.66	15.87	12.52	21.88	15.40	6.62	15.45	25.46	19.35	13.75	5.84	10.38	6.09	40.71	45.30	10.45	2.43	4.52
C18:3n-6 (γ-linolenic acid)	0.17	0.23	3.94[ǂ]	0.14	0.03	0.08	0.08	0.35	0.07	0.11	0.26	0.11	0.09	0.31	0.15	0.14	0.08	0.11	0.10	0.07	0.18	0.15	0.13	0.54	0.15	0.18	0.11	0.07	0.14	0.07	1.85	0.75	0.49	2.16	0.16
C20:4n-6 (arachidonic acid)	0.49	0.25	1.80	1.99	0.07	0.84	0.60	0.46	0.42	0.84	0.58	0.72	0.49	3.47	1.07	0.63	1.19	0.80	0.49	0.16	0.48	0.56	0.64	1.26	0.86	0.66	0.95	2.55	0.29	0.45	0.30	0.21	0.96	2.57	0.28
C22:4n-6 (docosatetraenoic acid)	0.09	0.13	3.32[ǂ]	0.23	0.16	0.14	0.07	0.13	0.04	0.04	0.12	0.06	0.12	0.02	0.05	0.15	0.06	0.21	0.14	0.09	0.13	0.09	0.09	0.58	0.13	0.09	0.03	0.26	0.07	0.11	0.09	0.09	0.54	0.95	0.26
**n-3 Polyunsaturated fatty acids**																																			
C18:3n-3 (α-linolenic acid)	2.01	1.30	1.70	2.59	4.01	6.85	0.66	4.23	1.34	0.62	0.82	0.96	0.32	0.87	1.17	1.20	2.13	2.58	2.96	2.89	4.99	2.15	2.48	1.09	2.37	2.38	1.31	0.27	0.51	0.21	0.77	0.25	5.85	1.89	0.58
C20:5n-3 (EPA)	0.79	0.62	0.66	1.94	0.28	1.95	0.10	0.13	0.20	0.06	0.08	0.10	0.09	0.01[†]	0.04	0.05	0.30	0.21	0.18	0.08	0.21	0.11	0.07	0.07	0.04	0.04	0.12	0.11	0.09	0.04	1.01	0.13	3.24	10.67	12.00
C22:5n-3 (DPA)	0.07	0.09	0.11	0.14	0.12	0.18	0.10	0.21	0.08	0.06	0.11	0.07	0.06	0.08	0.11	0.07	0.09	0.11	0.13	0.13	0.14	0.06	0.15	0.12	0.08	0.12	0.03	0.08	0.13	0.04	0.15	0.14	1.51	2.23	1.50
C22:6n-3 (DHA)	0.14	0.13	0.14	0.12	0.08	0.15[§]	0.06[§]	0.23	0.05	0.03	0.08	0.05	0.07	0.09	0.10	0.08	0.04	0.13	0.11	0.07	0.12[§]	0.08	0.09	0.10	0.09	0.14	0.05	0.09	0.16	0.08	3.65	0.21	4.28	13.52	6.39
**Summary fractions**																																			
SFA (% of total FA)	34.11	49.49	54.00	32.55	32.96	35.03	39.60	34.99	31.85	45.82	59.05	36.69	46.85	50.66	35.01	46.38	41.88	24.19	39.98	38.82	28.12	29.31	50.29	36.85	29.24	30.87	34.75	40.52	38.26	43.31	15.58	13.99	19.78	25.53	22.57
MUFA (% of total FA)	36.87	26.20	16.46	34.98	40.19	27.87	44.98	41.82	44.62	38.90	25.34	44.66	38.01	32.02	39.09	29.85	41.84	52.69	35.92	40.40	39.63	47.77	34.59	39.48	37.03	41.63	44.32	45.82	46.00	44.96	26.96	29.25	49.12	34.26	47.69
Σn-6 PUFA (% of total FA)	21.02	17.69	23.15	24.14	17.45	24.17	9.85	13.73	18.08	9.81	9.83	13.38	9.67	11.93	19.54	18.64	9.70	15.78	16.60	12.84	22.67	16.20	7.48	17.83	26.60	20.28	14.84	8.72	10.88	6.72	42.95	46.35	12.44	8.11	5.22
Σn-3 PUFA (% of total FA)	3.01	2.14	2.61	4.79	4.49	9.13	0.92	4.80	1.67	0.77	1.09	1.18	0.54	1.05	1.43	1.40	2.56	3.03	3.38	3.17	5.46	2.40	2.79	1.38	2.58	2.68	1.51	0.55	0.89	0.37	5.58	0.73	14.88	28.31	20.47
n-6/n-3 ratio	6.98	8.27	8.87	5.04	3.89	2.65	10.71	2.86	10.83	12.74	9.02	11.34	17.91	11.34	13.66	13.31	3.79	5.21	4.91	4.05	4.15	6.75	2.68	12.92	10.31	7.57	9.83	15.85	12.22	18.16	7.70	63.49	0.84	0.29	0.26

* FS5: a human food supplement under Directive 2002/46/EC; included for comparative purposes.

nd: not detected. Values are expressed as percentage of total peak area (%).

SFA, saturated fatty acids; MUFA, monounsaturated fatty acids; n-6 PUFA, n-6 polyunsaturated fatty acids; n-3 PUFA, n-3 polyunsaturated fatty acids; EPA, eicosapentaenoic acid; DPA, docosapentaenoic acid; DHA, docosahexaenoic acid; ALA, α-linolenic acid.

[ǂ]Unverified peak assignment; no declared feed ingredient provides a clear source that would explain the presence of this fatty acid at the reported proportion

[§]Unverified analytical observations

[†] Fatty acid values falling between the LOD (0.005%) and LOQ (0.015%) thresholds were fully detectable but should be interpreted as trace proportions due to the inherently higher measurement uncertainty be-low the quantification limit.
